# Transcriptome analysis of nitrogen assimilation preferences in *Burkholderia* sp. M6-3 and *Arthrobacter* sp. M7-15

**DOI:** 10.3389/fmicb.2025.1559884

**Published:** 2025-04-07

**Authors:** Ran Liu, Hongyi Qin, Qian Wang, Cheng Chu, Yunbin Jiang, Huan Deng, Cheng Han, Wenhui Zhong

**Affiliations:** ^1^College of Zhongbei, Nanjing Normal University, Danyang, Jiangsu, China; ^2^Jiangsu Provincial Key Laboratory of Materials Cycling and Pollution Control, School of Geographical Sciences, Nanjing Normal University, Nanjing, China; ^3^School of Environment, Nanjing Normal University, Nanjing, China; ^4^Jiangsu Center for Collaborative Innovation in Geographical Information Resource Development and Application, Nanjing, China

**Keywords:** inorganic nitrogen assimilation, nitrogen preference, bacterial strains, RNA-seq analysis, nitrogen metabolic pathway

## Abstract

**Introduction:**

Ammonium (NH_4_^+^) and nitrate (NO_3_^−^) are the two main forms of inorganic nitrogen (N) that exist in soil and both can be absorbed and utilized by plants. As a vast and crucial biome, soil microorganisms are responsible for mediating the inorganic N assimilation process and enhancing nitrogen use efficiency. Understanding how these microorganisms assimilate different forms of inorganic nitrogen is crucial. There are a handful of microorganisms that play a dominant role in the process of soil inorganic nitrogen assimilation and have a significant advantage in abundance. However, microbial preferences for ammonium or nitrate, as well as differences in their metabolic pathways under co-existing ammonium and nitrate conditions, remain unclear.

**Methods:**

In this study, two microbial strains with nitrogen assimilation advantages, *Burkholderia* sp. M6-3 and *Arthrobacter* sp. M7-15 were isolated from an acidic Chinese soil and then incubated by different sources of inorganic N to investigate their N preferences. Furthermore, RNA sequencing-based transcriptome analysis was used to map the metabolic pathways of the two strains and explore their explanatory potential for N preferences.

**Results:**

The results showed that strain M6-3 preferred to utilize NH_4_^+^ while strain M7-15 preferred to utilize NO_3_^−^. Although both strains shared similar nitrogen metabolic pathways, the differential expression of the glutamine synthetase-coding gene *glnA* played a crucial role in regulating their inorganic N preferences. This inconsistency in *glnA* expression may be attributed to *GlnR*, a global regulator of nitrogen utilization.

**Discussion:**

This research strengthens the theoretical basis for exploring the underlying causes of differential preferences for inorganic N forms and provided key clues for screening functional microorganisms to ultimately enhance inorganic nitrogen use efficiency.

## 1 Introduction

Nitrogen (N) is essential for all life on Earth (Stevens, 2019). Multiple forms of N are present in soil, including inorganic N [ammonium (NH_4_^+^) and nitrate (NO_3_^−^)] and low molecular weight organic N [such as amino acids and microbial biomass nitrogen (MBN)], which can be absorbed and utilized by plants (Beeckman et al., 2018). In agroecological systems, NH_4_^+^ and NO_3_^−^ are the most critical N sources for crops (Cui et al., 2017; Zhu Y. et al., 2021). Soil NO_3_^−^ and NH_4_^+^ concentrations are often insufficient for crop growth; applying additional N fertilizer to agricultural soils is a common practice to improve crop yields and ensure food security (Fageria and Baligar, 2005). Studies indicate that most crops exhibit differential preferences for inorganic N forms. For example, wheat and maize preferentially absorb NO_3_^−^ (Zhang et al., 2016), while rice shows a strong preference for NH_4_^+^ (Zhao et al., 2013). Similarly, soil microorganisms also display distinct inorganic N preferences. Microorganisms generally prefer to utilize NH_4_^+^ in soil (Christie and Wasson, 2001), possibly due to the lower energy requirement for NH_4_^+^ assimilation (Murphy et al., 2003). In contrast, other researchers found that even in alkaline soils with a high NH_4_^+^/NO_3_^−^ ratio, microbial assimilation of NO_3_^−^ remained dominant (Rochester et al., 1992). Certain studies have been conducted on the reasons for plants’ preference for NH_4_^+^ or NO_3_^−^, and it has been found that functional genes for ammonium and nitrate/nitrite transport may play a key role in their N preference (Hildebrand, 2005). However, the intrinsic causes of microbial preference for NH_4_^+^ or NO_3_^−^ are unclear.

Soil microorganisms are essential for ecosystem multifunctionality (Wagg et al., 2014). One of the underlying mechanisms is the assimilation process in which microorganisms absorb inorganic N, reducing their losses and enhancing N use efficiency (Zhu J. et al., 2021; Wang et al., 2024). The assimilation of inorganic N by soil microorganisms is strongly related to their N metabolic pathways (Pan et al., 2023). Numerous studies have investigated the assimilation processes of NH_4_^+^ and NO_3_^−^and found that NO_3_^−^ is the primary source of inorganic N used by most bacteria, yeasts, and algae (González et al., 2006; Wang and Song, 2024). NO_3_^−^ is reduced to NO_2_^−^ by assimilatory nitrate reductases (Nas) after being transported into the cell by nitrate transporters, followed by reduction to NH_4_^+^ by nitrite reductase (Jin et al., 2019). NH_4_^+^ is a crucial compound for nitrogen assimilation in many biological systems (Zhang L. J. et al., 2020) and can also be incorporated into the carbon skeleton through the glutamate dehydrogenase (GDH) or glutamine synthetase-glutamate synthase (GS-GOGAT) pathways (Mara et al., 2018; Gao et al., 2025) after entering the cell via ammonium transport family protein (Amt) transport (Andrade and Oliver., 2007; Williamson et al., 2022). In this series of processes, multiple functional genes for nitrogen metabolism are activated and expressed (Tu et al., 2019). After cells transport nitrate or nitrite into the cytoplasm through active transport by *NrT*/*NrTA*-regulated transporter proteins (Richardson et al., 2001), nitrate is reduced by *nasAB*-encoded Nas to nitrite, *nirBD*-encoded nitrite reductases reduce nitrite to ammonium (Zhang et al., 2015), and external nitrite can also be taken up and directly reduced to ammonium (Lledó et al., 2005). The ammonium produced by the nitrite reductase reaction is incorporated into the carbon skeleton via the GDH or GS–GOGAT pathways. The GDH and GS–GOGAT pathways are two pathways of ammonium assimilation, some microorganisms use only one of them strictly while others use both pathways (Gao et al., 2025). Thus, microbial utilization of inorganic N is closely related to its nitrogen metabolism pathways and functional gene expression. However, it is unclear whether microbial preference for inorganic N in the assimilation process is controlled by the difference in metabolic pathways or the expression level of functional genes mentioned above.

When adding multiple forms of N, either inorganic or organic, the majority of N was immobilized in microbial biomass (Kaštovská and Šantrůčková, 2011; Tahovská et al., 2013). Initially, all soil microorganisms were recognized to jointly participate in N assimilation and immobilization (Schimel, 1995). Recent studies proposed that N assimilation is performed by a relatively select group of microorganisms rather than a large number of seemingly redundant groups of microbial taxa (McGuire and Treseder, 2010). This finding was further supported by the fact that only a few groups of microorganisms contributed to 82–88% of the assimilated N in meadow soil, using a quantitative ^15^N-labeled stable isotope probing approach (Dong et al., 2022; Morrissey et al., 2018), and also evidenced in shelf environments (Wawrik et al., 2012). Therefore, it is feasible to isolate dominant N-assimilating strains that prefer different inorganic N sources, which can facilitate the study of their assimilation differences.

This study aims to address the following issues in microbial nitrogen metabolism research: (1) the molecular mechanisms underlying the differential preferences for inorganic nitrogen in two soil bacteria; and (2) the differences in nitrogen utilization regulation of the two bacteria when different forms of inorganic nitrogen serve as nitrogen sources. Using *Burkholderia* sp. M6-3 and *Arthrobacter* sp. M7-15 isolated from acidic upland soils, we sought to: (1) Characterize strain specific nitrogen source preferences through phenotypic analysis; (2) Elucidate the differences in dynamic gene expression under mixed nitrogen conditions through transcriptomic analysis; and (3) Identify conserved regulatory networks and strain specific molecular switches that control nitrogen metabolism. These integrated approaches are aimed at understanding microbial nitrogen utilization strategies and providing a theoretical basis for improving nitrogen use efficiency in agricultural systems.

## 2 Materials and methods

### 2.1 Strains and growth conditions

**Figure 1:**
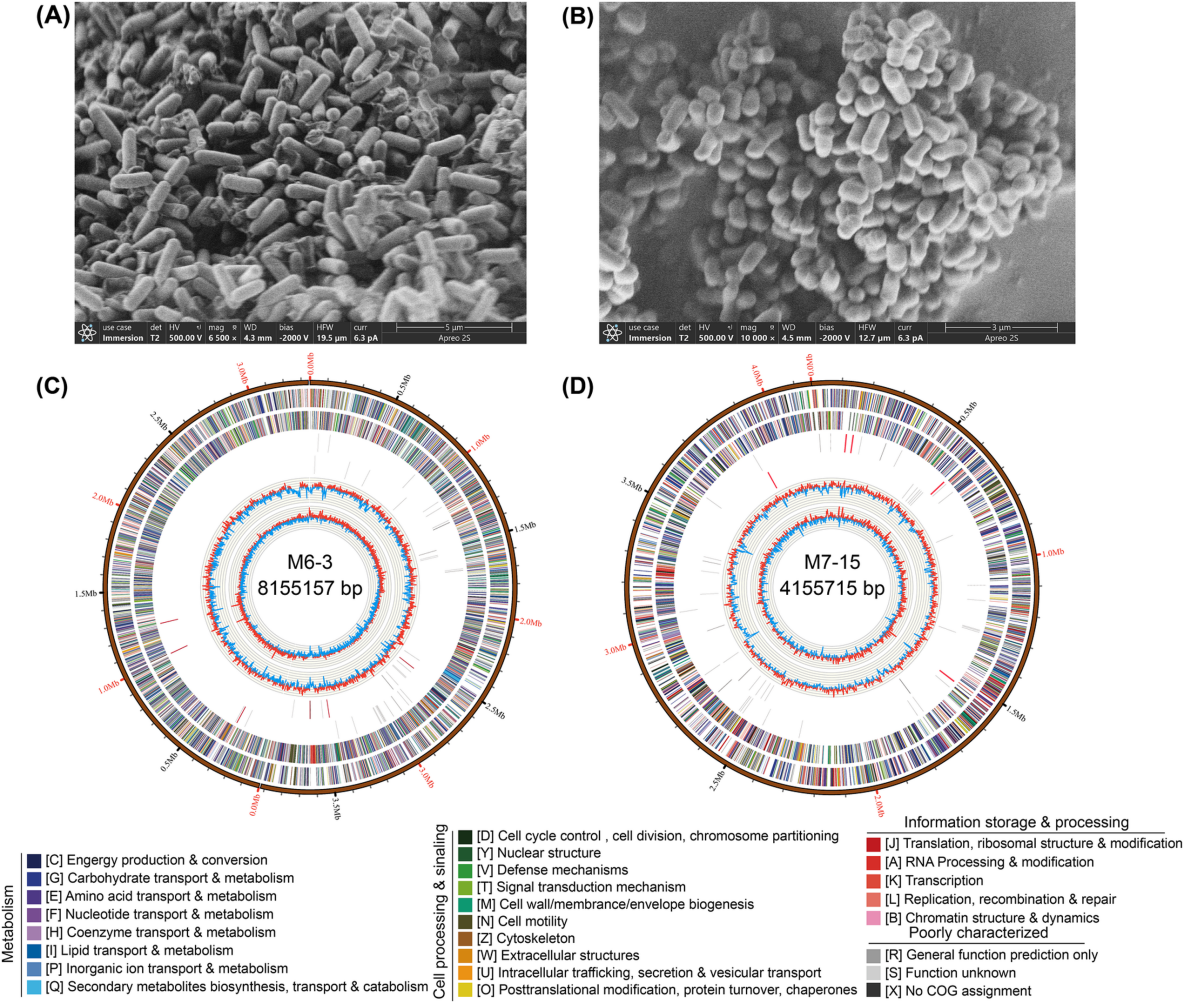
Morphology and genome information of *Burkholderia* sp. M6-3 **(A,C)** and *Arthrobacter* sp. M7-15 **(B,D)**. The circular genome map is displayed from the outer circle to innermost, as follows: scale marks of the genome; protein-coding genes on the forward strand; protein-coding genes on the reverse strand; tRNA (black) and rRNA (red) genes on the forward strand; tRNA (black) and rRNA (red) genes on the reverse strand; GC content; GC skew. Protein-coding genes are color coded according to their COG categories.

The test strains *Burkholderia* sp. M6-3 and *Arthrobacter* sp. M7-15 (Figure 1) were isolated from an acidic upland soil (Liu et al., 2023), which came from a long-term field fertilization experiment site (28°15′30″N, 116°20′24″E) started in 1986 at the Jiangxi Institute of Red Soil and Germplasm Resources, China. The area has a subtropical monsoon climate with an average annual rainfall of 1,537 mm and an average annual temperature of 17.5°C. The soil is derived from Quaternary red clay, and the cropping system is the spring corn-autumn corn-winter idle system. Soil sampling was conducted in three plots. Surface soil (0–15 cm) was collected using a five-point sampling method from each plot, mixed thoroughly, and sieved through a 2 mm mesh. Three replicates of soil samples were collected. Soil pH was 4.90, soil electrical conductivity was 27.20 μS cm^−1^, organic carbon content was 7.14 g kg^−1^, and total nitrogen was 0.92 g kg^−1^ (Zhong et al., 2010; Wang et al., 2015).

The media required for the strain’s growth were prepared as follows: liquid nutrient agar medium (NA medium) (Mishra, 2019): 10 g peptone, 3 g beef extract and 5 g NaCl, dissolved in 1000 mL of water (pH 7.0), and autoclaved at 121°C for 20 min. Nitrogen-free medium (NF medium) (Patil et al., 2020): 10 g glucose, 0.2 g K_2_HPO_4_, 0.2 g MgSO_4_, 0.2 g NaCl, 0.1 g K_2_SO_4_, 1.0 g CaCO_3_, dissolved in 1000 mL of water (pH 7.0), autoclaved at 121°C for 20 min (glucose alone autoclaved at 115°C for 30 min).

Inorganic nitrogen at 4 μmol N g^−1^ soil and glucose at 500 μg C g^−1^ soil were added to activate the soil nitrogen assimilation and enrich the N-assimilating microorganisms (Morrissey et al., 2018). The dominant microbial groups with the highest relative abundance were obtained by high-throughput sequencing of total soil DNA, and individual strains were isolated from the NF medium coated with soil suspension. A total of 154 isolated bacteria were incubated. Bacterial genomic DNA was extracted using the Rapid Bacterial Genomic DNA Isolation Kit (Sangon Biotech). A 25 μL PCR reaction mixture was employed to amplify the selected suspected strains, consisting of 12.5 μL of PCR Master Mix, 0.5 μL of each primer (27F: 5′-AGAGTTTGATCCTGGCTCAG-3′, 5 μM; 1492R: 5′-TACGGYTACCTTGTTAYGACTT-3′, 5 μM) 10.5 μL of ddH₂O, and 1 μL of DNA template (Haas et al., 2011). The amplification conditions were as follows: 94°C for 5 min; 35 cycles of denaturation at 94°C for 30 s, annealing at 58°C for 30 s, and extension at 72°C for 90 s; followed by a final extension at 72°C for 7 min. The PCR products were analyzed by agarose gel electrophoresis to assess quantity and quality. Sequencing was performed by Sangon Biotech (Shanghai) Co., Ltd. (Shanghai, China) using an Applied Biosystems™ 3730XL Genetic Analyzer (Thermo Fisher Scientific) with bidirectional sequencing. Raw sequencing data were edited and assembled using Sequencher 5.0 software (Gene Codes Corporation). The resulting sequences were subjected to BLAST alignment against the NCBI GenBank database. The sequences of the strains were compared to the sequences of the dominant microorganisms in the soil, then the strains *Burkholderia* sp. M6-3 and *Arthrobacter* sp. M7-15, which had the closest sequence, was isolated.

### 2.2 Whole genome sequencing of strains

Both strains were sequenced at Guangdong Magigene Biotechnology Co., Ltd. (Guangzhou, China) on the Illumina Hiseq1500 platform. For the libraries that passed the quality inspection, paired-end 150 bp (PE150) sequencing was performed on an on the Illumina Hiseq 1,500 platform. Sequencing data were filtered and assembled using a *de novo* assembly strategy (Metzker, 2010). Pure third-generation sequencing (TGS) data were assembled with SMRT Link v5.1.0 (Jayakumar and Sakakibara, 2019), while hybrid assembly integrating second- and third-generation sequencing data was performed with Unicycler (Wick et al., 2017). Genomic data of the strains were annotated by the KEGG (Kyoto Encyclopedia of Genes and Genomes) (Kanehisa and Goto, 2000), and a genome-wide Blast^1^ similarity search (*E-value* < 1^−10^, minimal alignment length percentage > 40%) was performed against on databases, including COG, KEGG, GO, NR, and Swiss-Prot (Pan et al., 2017; Sun et al., 2019). Sequencing data were used to map the nitrogen metabolism pathway and identify functional genes based on KEGG annotation. The raw genomic data were deposited in the NCBI Sequence Read Archive database under the accession number PRJNA891540.

### 2.3 Strain growth under different inorganic nitrogen sources

Strains of *Burkholderia* sp. M6-3 and *Arthrobacter* sp. M7-15 were inoculated into the NA medium and incubated at 200 r/min 30°C until the proliferation phase, and the bacteria were collected, washed three times with saline, and resuspended.

Four treatments, nitrogen-free medium (Control) and with additions of KNO_3_ (NO_3_^−^), NH_4_Cl (NH_4_^+^) and KNO_3_ + NH_4_Cl (NO_3_^−^ + NH_4_^+^), were considered. The concentration of each nitrogen source in the medium was made at 100 mg N L^−1^ (pH = 7.0 ± 0.2). The parameters of the culture medium were determined based on the pre-experiment (Supplementary Figures 1, 2). Each treatment was autoclaved at 121°C for 20 min, injected with 100 μL of resuspended bacteria and incubated at 30°C 200 r/min. All treatments were repeated at least thrice. The biomass accumulation was determined for every 6 h by measuring the optical density at 600 nm (OD_600_) using a BioTek Synergy™ HT Multidetection microplate reader. Ammonium, nitrite and nitrate contents were measured with a San^++^ continuous flow analyzer (Skalar, Breda, Netherlands). Microbial biomass nitrogen (MBN) was determined by collecting all the bacteria in the medium, freeze-drying them, using a Microwave Digestion System (TANK 40, SINEO, Shanghai, China), and then using a total organic carbon analyzer (TOC-L CPH, Shimadzu, Japan).

### 2.4 Transcriptome analysis by RNA sequencing

For each treatment, bacteria were collected by centrifugation at 6000 r/min for 5 min during the proliferation phase and stored at −80°C refrigerator after quick-freezing with liquid nitrogen. Total RNA was extracted from the collected organisms using the TRIzol method. Quality checks of extracted RNA samples were conducted using a NanoDrop One Spectrophotometer (Thermo Scientific, Waltham, MA, United States) and Agilent 4,200 Tape Station (Santa Clara, CA, United States). Ribosomal RNA (rRNA) was removed from the total RNA using an Epicenter Ribo-Zero rRNA Removal Kit (Madison, WI, United States). Each treatment included 3 replicates, and the extracted RNAs were pooled in equal proportions for sequencing. RNA sequencing was performed by Guangdong Magigene Biotechnology Co., Ltd. (Guangzhou, China).

Library preparation was carried out with a NEBNext^®^ Ultra II™ Directional RNA Library Prep Kit (Illumina, San Diego, CA, United States) according to the standard protocol. The library was subjected to quality control, and PE150 sequencing was performed using the Illumina high-throughput sequencing platform. The raw data files obtained from sequencing were transformed into raw sequencing sequences (Raw Reads) by Base Calling (Ledergerber and Dessimoz, 2011), and the results were stored in FASTQ (fq) file format. Sequence quality control and data filtering was performed with fastp (Chen et al., 2018), and rRNA reads were filtered out before analysis. The ribosome contamination was removed by Bowtie 2 (Langmead and Salzberg, 2012), and the sequencing quality was analyzed by RSeQC (Wang et al., 2012). The read count was calculated using RSEM (Li and Dewey, 2011). The read counts of transcripts were normalized using the “tximport” package (Soneson et al., 2015) to calculate Fragments Per Kilobase of transcript per Million mapped reads (FPKM), which can eliminate the effects of gene length and differences in sequencing depth on the calculation of gene expression. GFOLD (v1.1.4) was employed to identify differentially expressed genes (DEGs) across different inorganic N treatments (Feng et al., 2012). GFOLD ranks differentially expressed genes from RNA-seq data, relying on the posterior distribution of log fold change, to overcome the limitations of *p-value*, providing stable and biologically relevant results (Takahashi et al., 2022). The significance cutoff was set at 0.01 (−sc 0.01), and a |GFOLD value| > 1 was also required for 2-fold change or greater (Yang et al., 2017; Adachi et al., 2024). The genes were annotated using the KEGG database. After annotating each gene into a KEGG functional category, and comparisons were made in terms of that functional category (Guo et al., 2005). RNA-seq data have been submitted to the NCBI Sequence Read Archive (SRA) under the BioProject accession PRJNA891352.

### 2.5 Statistical analysis

Statistical analyses were conducted using SPSS (V20.0, IBM, United States). The one-way analysis of variance (ANOVA) was employed to assess the significant differences in OD_600_, inorganic nitrogen content and biomass among treatments (*p* < 0.05, *n* = 3, Duncan’s test).

## 3 Results

### 3.1 Strain morphology and genomic characteristics

Visualized on solid medium, both strains, *Burkholderia* sp. M6-3 and *Arthrobacter* sp. M7-15 formed moist, white, and round colonies with neatly defined edges and raised centers. The diameter of each colony is approximately 1.0 mm. When observed using scanning electron microscopy (SEM), *Burkholderia* sp. M6-3 exhibits a rod shape, measuring 0.6–0.7 μm × 1.0–2.2 μm (Figure 1A), while *Arthrobacter* sp. M7-15 also displays a rod shape, measuring 0.4–0.5 μm × 0.8–1.2 μm (Figure 1B).

The whole genome maps of two strains were visualized using Circos. Each genome comprises both coding and non-coding regions that are essential for transcriptional regulation, translational regulation, epigenetic functions, and other biological processes. The genome size of strain M6-3 was 8,155,157 bp, with a GC content of 61.6%. This strain contained two chromosomes and two plasmids (Figure 1C). The total number of coding genes was 7,989, and the length of coding regions accounted for 83.53% of the genome. The non-coding RNA repertoire included 63 tRNAs, 18 rRNAs, and 5 sRNAs (Supplementary Table 1). In contrast, the genome size of strain M7-15 was 4,155,715 bp, with a GC content of 63.31%. This strain contained one chromosome (Figure 1D). The number of coding genes was 4,092, with coding regions constituting 87.95% of the genome. The non-coding RNA in this strain included 57 tRNAs and 15 rRNAs (Supplementary Table 1).

### 3.2 Preference for inorganic nitrogen of two strains

**Figure 2:**
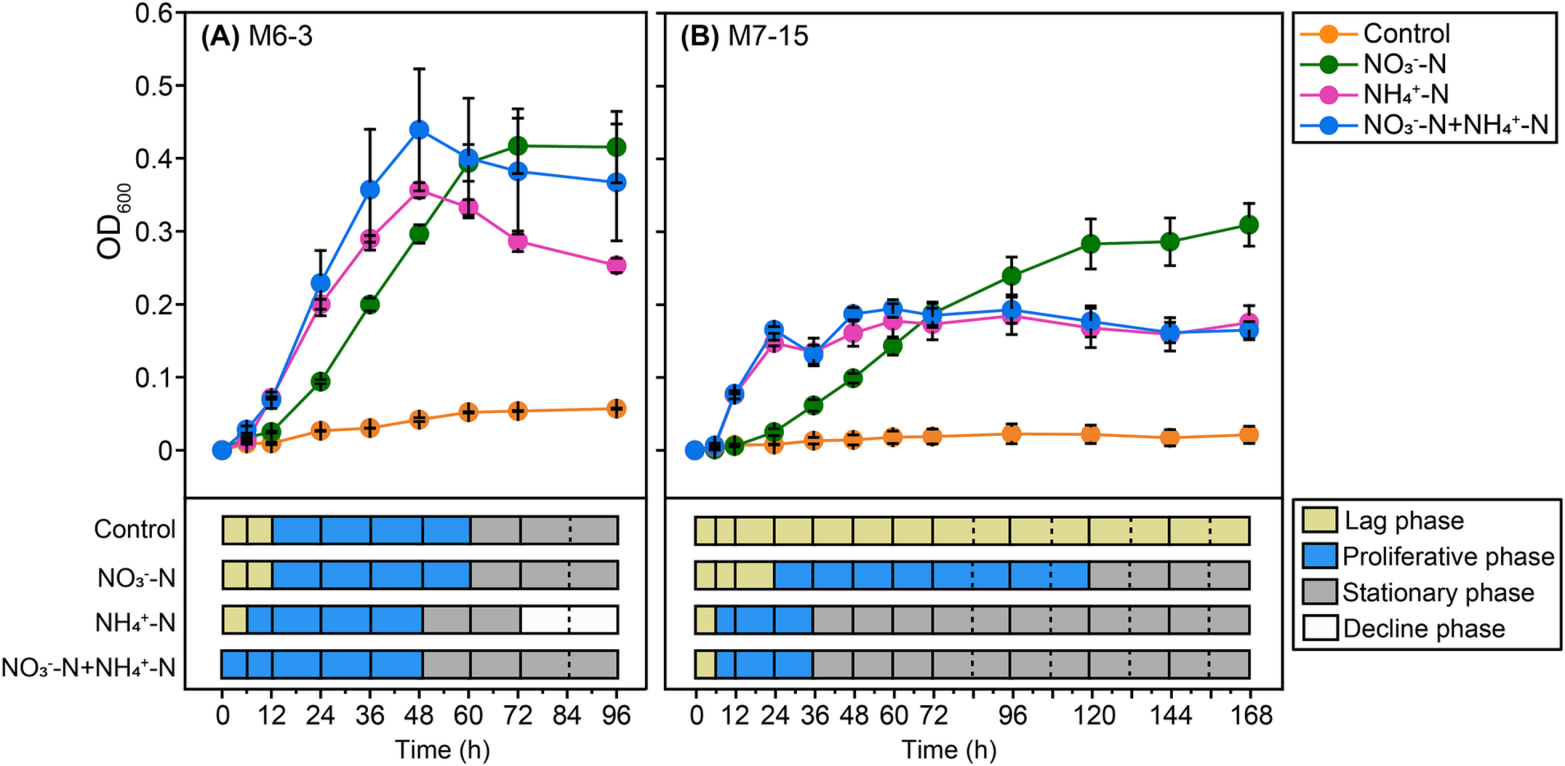
Growth of *Burkholderia* sp. M6-3 **(A)** and *Arthrobacter* sp. M7-15 **(B)** under different inorganic nitrogen application. Linear graphs indicate the growth curve of the bacterial strains in terms of the change in OD_600_ value. The time axis indicates the different time periods of bacterial strain growth. The different periods of strains growth were classified according to whether there was a significant difference between their OD_600_ values (*p* < 0.05, *n* = 3, Student’s *T*-test). Control refers nitrogen-free treatment. Bars indicate the standard deviation (*n* = 3).

The growth characteristics of both strains were assessed using OD_600_ at various sources of inorganic nitrogen. Both M6-3 and M7-15 exhibited relatively longer lag and exponential growth phases in response to NO_3_^−^ treatment compared to the treatments with NH_4_^+^ and NO_3_^−^ + NH_4_^+^ (Figure 2). For the NO_3_^−^ + NH_4_^+^ treatment, the actual lag phase may be less than 6 h, indicating that the response of both strains to NH_4_^+^ is more rapid than to NO_3_^−^. While the growth curves of both strains did not show statistically significant differences between the NH_4_^+^ and NO_3_^−^ + NH_4_^+^ treatments, significant differences were observed between the NO_3_^−^ and NH_4_^+^ or NO_3_^−^ + NH_4_^+^ treatments (*p* < 0.05) (Figure 2).

**Figure 3:**
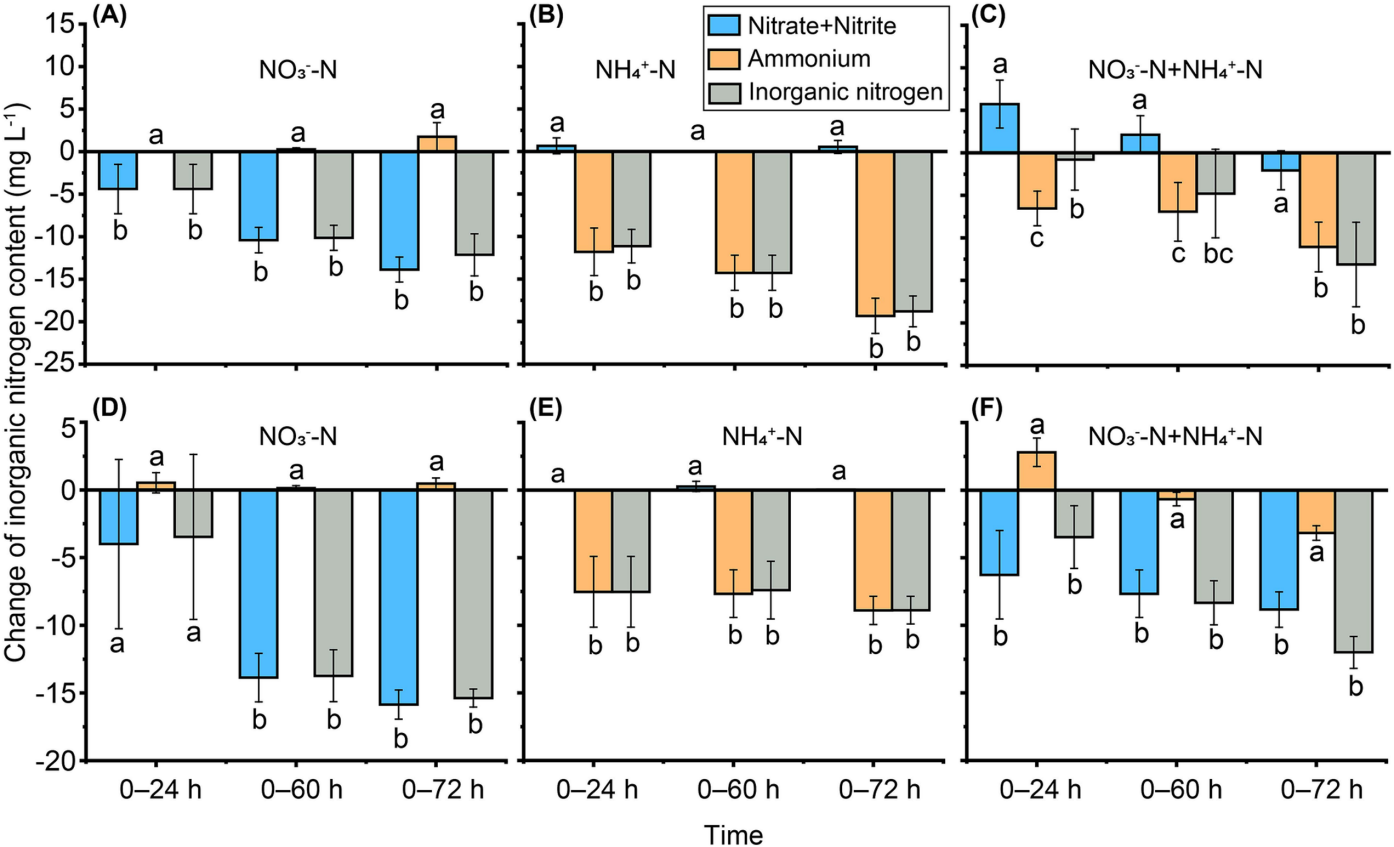
Changes in inorganic nitrogen contents during 72 h of incubation with *Burkholderia* sp. M6-3 **(A–C)** and *Arthrobacter* sp. M7-15 **(D–F)**. “0–24 h,” “0–60 h” and “0–72 h” indicate the changes of inorganic nitrogen content from 0 h to 24 h, from 0 h to 60 h and from 0 h to 72 h, respectively. Bars indicate the standard deviation, different letters indicate significant differences (*p* < 0.05, *n* = 3, Duncan’s test).

For strain M6-3, the measured nitrate content exhibited a decrease of 4.40 ± 2.90 mg L^−1^ over the 24 h period, and a decrease of 13.87 ± 1.47 mg L^−1^ over the 72 h period in the NO_3_^−^ treatment. The measured ammonium content decreased by 11.80 ± 2.79 mg L^−1^ during the 24 h interval and by 19.33 ± 2.07 mg L^−1^ during the 72 h interval in the NH_4_^+^ treatment. The decrease in nitrate content observed in the NO_3_^−^ treatment was significantly smaller than those noted for ammonium content in the NH_4_^+^ treatment. In the mixed nitrate and ammonium N treatment, total inorganic N decreased by 13.13 ± 4.97 mg L^−1^, with nitrate and ammonium contributing 2.06 ± 2.28 mg L^−1^ and 11.07 ± 2.93 mg L^−1^, respectively (Figures 3A–C). These findings indicate that strain M6-3 preferentially utilizes ammonium N.

For strain M7-15, the measured nitrate content exhibited a decrease of 4.00 ± 10.83 mg L^−1^ over a 24 h period, and a decrease of 15.85 ± 1.07 mg L^−1^ over the 72 h period in the NO_3_^−^ treatment. The measured ammonium content decreased by 7.53 ± 2.61 mg L^−1^ during the 24 h interval and by 8.90 ± 1.04 mg L^−1^ during the 72 h interval in the NH_4_^+^ treatment. The decrease in nitrate content observed in the NO_3_^−^ treatment was significantly greater than the decrease in ammonium content noted in the NH_4_^+^ treatment. In the mixed treatment of nitrate and ammonium nitrogen, the total inorganic nitrogen decreased by 12.00 ± 1.18 mg L^−1^, with contributions from nitrate and ammonium amounting to 8.83 ± 1.31 mg L^−1^ and 3.17 ± 0.54 mg L^−1^, respectively (Figures 3D–F). These findings indicate that the strain M7-15 preferentially utilizes nitrate nitrogen.

### 3.3 Expression profiles of nitrogen metabolism-related genes

**Figure 4:**
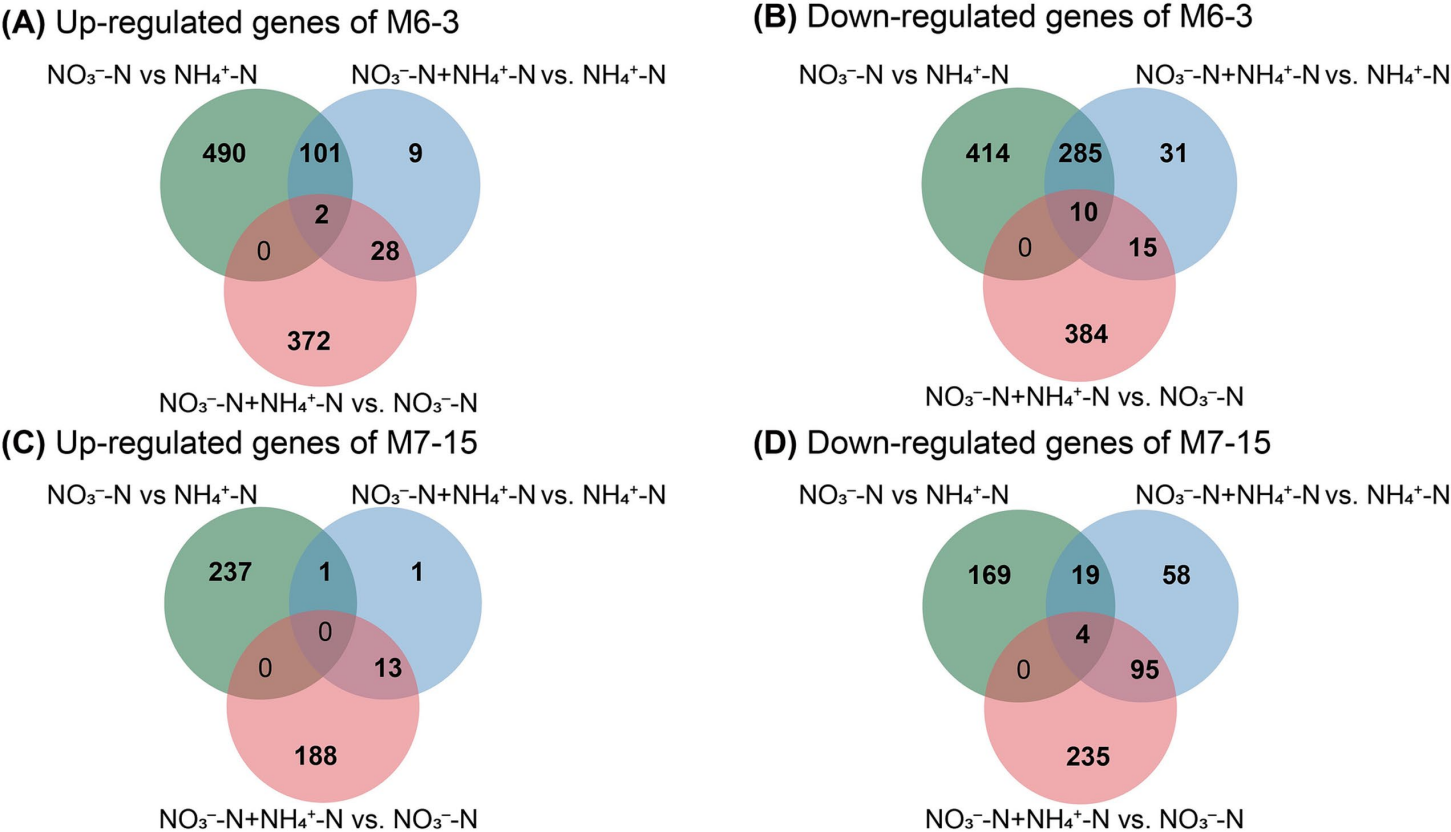
The intersections of differentially expressed genes (DEGs) of *Burkholderia* sp. M6-3 **(A,B)** and *Arthrobacter* sp. M7-15 **(C,D)** under different inorganic nitrogen treatments.

**Figure 5:**
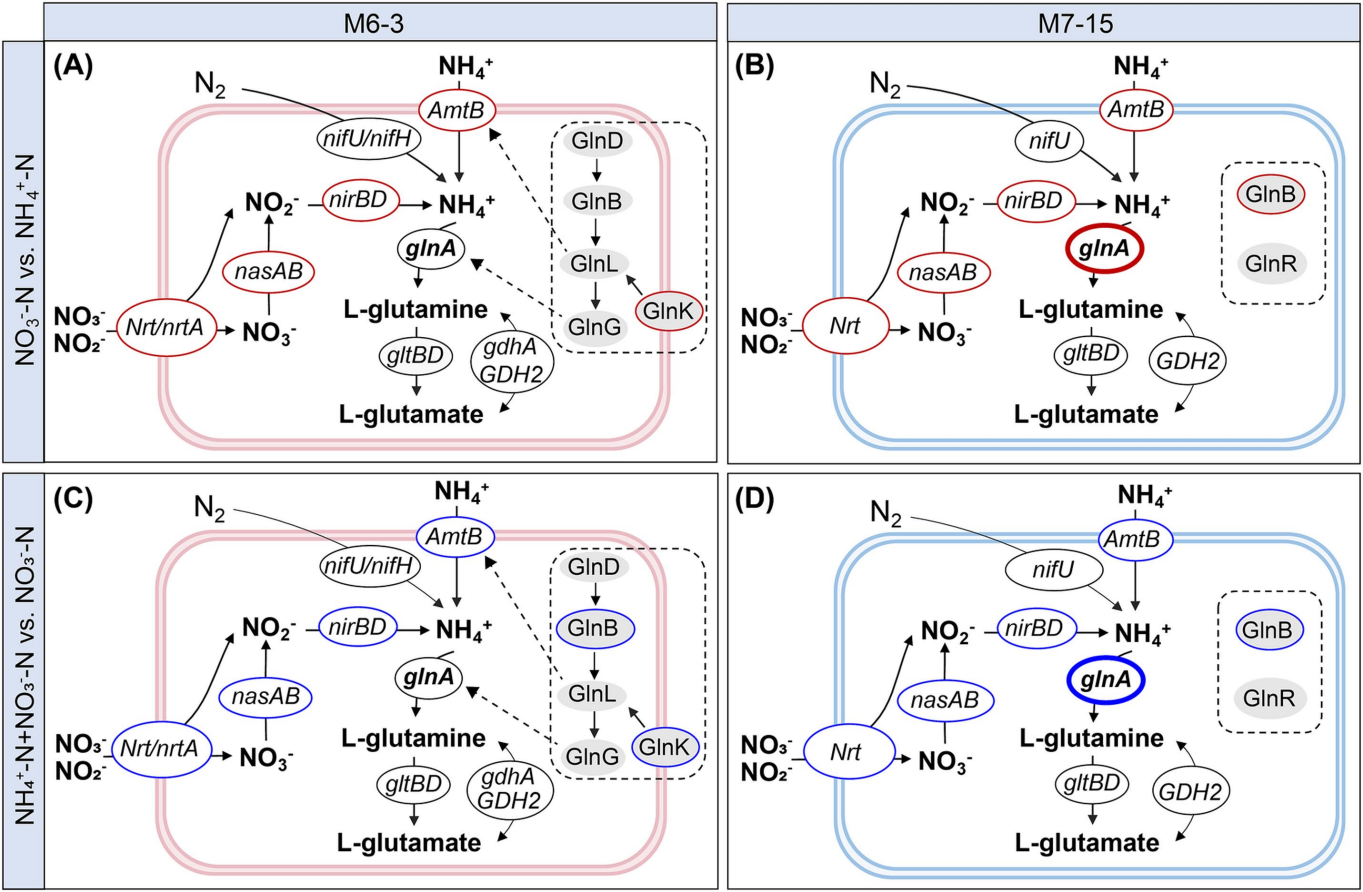
Differential transcript expression of functional genes for nitrogen metabolism in ammonium nitrogen, nitrate nitrogen and mixed nitrogen sources by *Burkholderia* sp. M6-3 **(A,C)** and *Arthrobacter* sp. M7-15 **(B,D)**. The significance cutoff was set at 0.01 (−sc 0.01), and a |GFOLD value| > 1 was also required for 2-fold change or greater. Red borders indicate genes significantly up-regulated (Gfold >1); blue borders indicate genes significantly down-regulated (Gfold < −1), and black borders indicate no significant difference. Gray ovals indicate the nitrogen regulatory factors associate with the two-component regulatory system.

For strain M6-3, a total of 1,302 DEGs was identified from the sample groups NO_3_^−^-N vs. NH_4_^+^-N, of which 593 were significantly up-regulated and 709 were significantly down-regulated (Figures 4A,B). In the sample groups NO_3_^−^-N vs. NH_4_^+^-N for strain M7-15, a total of 430 DEGs were identified, with 238 significantly up-regulated and 192 significantly down-regulated (Figures 4C,D). Following KEGG annotation, 13 and 10 types of genes were selected to map nitrogen metabolic pathways for strains M6-3 and M7-15, respectively. The results indicated that both M6-3 and M7-15 shared a similar nitrogen metabolic pathway, except for the NtrC family genes in the two-component system (Figure 5). After nitrate is transported into the cell via *NrT/NrTA*-regulated transporter proteins, it is reduced to nitrite by Nas, then reduced to NH_4_^+^ by nitrite reductase, and then incorporated into the carbon skeleton by GDH or GS-GOGAT pathway. Based on the mapped nitrogen metabolism pathway, it is feasible to investigate the underlying causes of the differential preferences for inorganic N.

From the sample groups NO_3_^−^-N vs. NH_4_^+^-N, the nitrate/nitrite transport and reduction genes *Nrt*/*NrtA*, *nasAB*, *nirBD*, the ammonium transporter *AmtB* and the nitrogen status sensor *GlnB*/*GlnK* were significantly up-regulated in both strains M6-3 and M7-15. However, the glutamine synthetase-coding gene *glnA* was significantly up-regulated only in strain M7-15, while no significant change was observed in M6-3 (Figures 5A,B). In the sample groups NH_4_^+^-N + NO_3_^−^-N vs. NO_3_^−^-N, the genes *Nrt*/*NrtA*, *nasAB*, *nirBD*, *AmtB,* and *GlnB*/*GlnK* were significantly down-regulated for both strain M6-3 and M7-15. Additionally, the *glnA* gene showed significant down-regulation only in M7-15 but not in M6-3 (Figures 5C,D). In the sample groups NH_4_^+^-N + NO_3_^−^-N vs. NH_4_^+^-N, the gene *GlnB* in M6-3 and the gene *nirBD* in M7-15 were significantly down-regulated, while the other genes had no significant differences (Supplementary Figure 3).

## 4 Discussion

### 4.1 Response of two bacterial strains to different inorganic nitrogen

This study found that M6-3 and M7-15 exhibited longer lag phases and exponential growth phases under NO_3_^−^-N treatment (Figure 2), which might be related to the metabolic mechanism of nitrate N. The utilization of nitrate N usually requires its reduction to ammonium N through the nitrate reductase system, a process that may involve energy consumption and the induction of gene expression, thereby leading to an extended lag phase (Wu et al., 2016). Notably, the lag phases of both strains were shortened under NO_3_^−^-N + NH_4_^+^-N mixed treatment (Figure 2), suggesting that the presence of NH_4_^+^-N might accelerate growth initiation through a synergistic effect or metabolic shortcut. This phenomenon is consistent with the reported characteristics of certain strains that preferentially utilize ammonium nitrogen (Geisseler et al., 2010). Although there was no statistical difference in the growth curves between NH_4_^+^-N and NO_3_^−^-N + NH_4_^+^-N treatments for both strains, the biomass was slightly higher under NO_3_^−^-N + NH_4_^+^-N treatment compared to pure NH_4_^+^-N conditions, which might reflect that diverse nitrogen forms are beneficial for increasing gene richness and thus promoting microbial growth (Ameer et al., 2025). However, the specific molecular mechanism underlying this difference requires further experimental verification.

The strains showed different preferences for different inorganic nitrogen. Inoculated with M6-3, the reduction of ammonium N was 1.4 times of the reduction of nitrate N under the NH_4_^+^-N or NO_3_^−^-N treatment (19.33 ± 2.07 mg L^−1^ vs. 13.87 ± 1.47 mg L^−1^), and ammonium N reduction was 5.4 times that of nitrate N reduction under the NO_3_^−^-N + NH_4_^+^-N treatment (11.07 ± 2.93 mg L^−1^ vs. 2.06 ± 2.28 mg L^−1^) during 0–72 h. Inoculated with M7-15, the reduction of nitrate N was 1.8 times greater than the reduction of ammonium N under the NH_4_^+^-N or NO_3_^−^-N treatment (15.85 ± 1.07 mg L^−1^ vs. 8.90 ± 1.04 mg L^−1^), and 2.8 times greater than the reduction in ammonium N under the NO_3_^−^-N + NH_4_^+^-N treatment (8.83 ± 1.31 mg L^−1^ vs. 3.17 ± 0.54 mg L^−1^) during 0–72 h. The results indicated that the M6-3 preferred to absorb ammonium N, and M7-15 preferred to absorb nitrate N (Figure 3). Previous studies on inorganic N utilization have often found that ammonium N is more preferentially immobilized by microorganisms than nitrate N (Christie and Wasson, 2001; Geisseler et al., 2010), possibly because the lower energy requirement for ammonium N assimilation into microbial cells (Murphy et al., 2003), and possibly due to ammonium N can inhibit nitrate N assimilation. Thus, ammonium appears to be a more readily available direct source of nitrogen for microorganisms, while the corresponding nitrate is more difficult to utilize directly. But some studies have revealed a higher microbial demand for nitrate in grassland and forest soil, which may be attributed to nitrogen limitation resulting from low NH_4_^+^ concentrations in the soil (Hatch et al., 2000; Song et al., 2025). In the absence of or low levels of ammonium concentration within the growth environment, bacterial nitrate removal rates can reach up to 82% (Burger and Jackson, 2003; Huang et al., 2021).

Previous research has demonstrated that the preference of microorganisms for ammonium or nitrate is contingent upon their species and growth conditions (Bengtsson et al., 2003; Huang et al., 2021; Li et al., 2017). In addition to growth conditions, changes in the strains themselves will have a more direct impact on their preference for ammonium or nitrate, so the study investigates the underlying reasons for this preference by examining the characteristics and gene transcription differences among microbial strains.

### 4.2 Transcriptional difference and metabolic mechanism of two strains under different inorganic nitrogen

**Figure 6:**
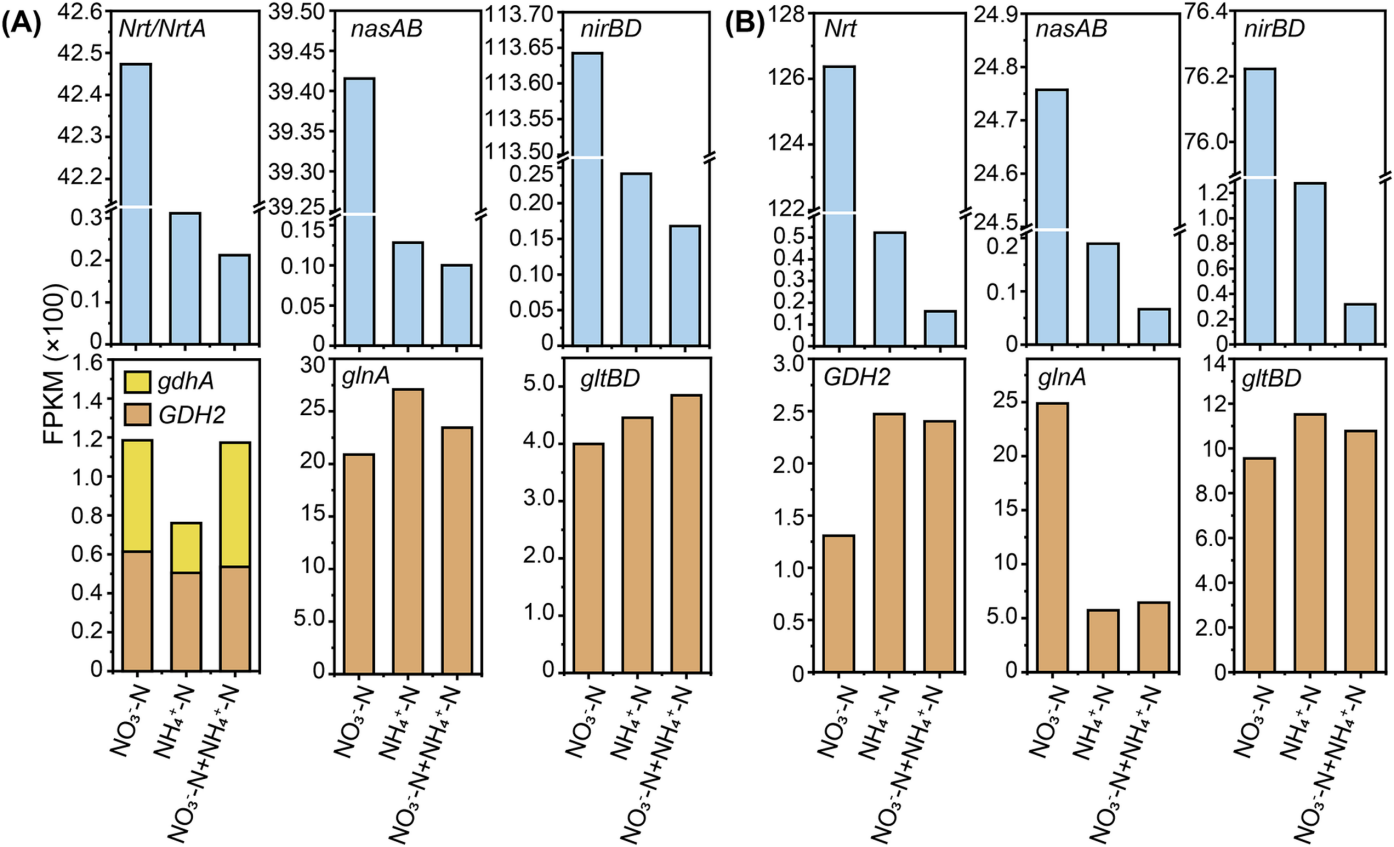
The expression levels of major nitrogen metabolism genes in *Burkholderia* sp. M6-3 **(A)** and *Arthrobacter* sp. M7-15 **(B)** under different inorganic nitrogen treatment.

The nitrogen metabolism pathways were similar between *Burkholderia* sp. M6-3 and *Arthrobacter* sp. M7-15, except for the NtrC family genes in a two-component system, which serves as the regulators of nitrogen assimilation (North et al., 2023). Both strains contained nitrate/nitrite transport and reduction genes and organic substance degradation and synthesis genes, including *NrT*/*NrTA*, *nasA*/*B*, *nirB*/*D*, *gdhA*/*GDH2*, *glnA*, and *gltB*/*D* (Tu et al., 2017; Tu et al., 2019). In this study, nitrate/nitrite is converted to NH_4_^+^ through transport and reduction regulated by the genes *NrT*/*NrTA*, *nasA*/*B*, and *nirB*/*D*. Additionally, NH_4_^+^ can also be directly transported into the cell via *AmtB*. In the NH_4_^+^ assimilation pathway, it was found that the expression of genes encoding GDH was generally at a low level, and both M6-3 and M7-15 mainly used the GS–GOGAT pathway for ammonium utilization rather than the GDH pathway (Figure 6), even though more energy expenditure required (Wu et al., 2016). In addition, under ammonium-limited conditions, the ammonium transporter (*AmtB*) can capture extracellular NH_4_^+^ into the cell through active transport, but this process is subject to strict regulation by the nitrogen regulatory system (Liu et al., 2020).

The presence of NH_4_^+^ significantly repressed the expression of nitrate and nitrite transport and reduction genes, and the ability to utilize nitrate of microbial cells under high concentrations of NH_4_^+^ will be greatly repressed (Flores et al., 2005; Martínez-Espinosa et al., 2007). In both strains M6-3 and M7-15, compared to NH_4_^+^-N treatments, genes *Nrt*/*NrtA*, *nasAB*, and *nirBD* were significantly up-regulated in treatments where only NO_3_^−^-N was present (Figures 5A,B), and they were significantly down-regulated in NO_3_^−^-N + NH_4_^+^-N treatment compared to NO_3_^−^-N treatment (Figures 5C,D). In M6-3, NH_4_^+^ severely inhibited the expression of the genes for nitrate utilization in the treatment of ammonium-nitrate coexistence, while no other response occurred for other nitrogen assimilation genes, which resulted in a preferential utilization of ammonium over nitrate by M6-3.

However, different from M6-3, in the case of the same inhibition by NH_4_^+^, the glutamine synthetase gene *glnA* in M7-15 gave feedback to this inhibition, causing this strain to assimilate more nitrate. Gene *glnA* in M7-15 was significantly up-regulated in NO_3_^−^-N treatment compared with NH_4_^+^-N (Figure 5B), which was significantly down-regulated in NO_3_^−^-N + NH_4_^+^-N treatment compared with NO_3_^−^-N (Figure 5D). This may be one of the reasons why the assimilation of M7-15 to nitrate N is higher than that to ammonium N. The *glnA* gene encodes glutamine synthetase, which is the central enzyme for nitrogen assimilation. Glutamine is a significant nitrogen source that is required for the organism (Zhang L. J. et al., 2020; Zhang W. et al., 2020), which not only provides glutamine for biosynthesis, but also cooperates with GOGAT to assimilate ammonium (Martínez-Espinosa et al., 2006). Studies have shown that glutamate synthetase and glutamine synthetase enabled the growth of strain in a medium with low ammonium concentration or only nitrate as a single nitrogen source (Pire et al., 2014), and *glnA* and *gltBD* genes were important genes for the growth of the strain in pure nitrate nitrogen medium. Therefore, the transcriptional expression of the strains themselves was an important factor in their response to the variable environment (Wang et al., 2022), and nitrogen-assimilating strains show different preferences in the face of different inorganic nitrogen sources by regulating the transcriptional expression of their functional genes. The *glnA* gene was significantly up-regulated in NO_3_^−^-N treatment, resulting in more nitrogen sources being utilized. Other researchers have also found a significant advantage in the expression of the *glnA* gene within a nitrate-preferred strain of *Bacillus megaterium* (You et al., 2022). The gene *glnA* seems to be the key factor for the difference in ammonium or nitrate preferences by strain M7-15. The difference in gene *glnA* expression in the two strains might be related to the nitrogen two-component system, and further investigation revealed a large difference in the nitrogen regulatory factors of the two strains.

Within the NtrB-NtrC two-component system, abundant nitrogen regulatory factors annotated to M6-3, including GlnD, *GlnB*, *GlnK*, GlnL (ntrB), and GlnG (ntrC), among which *GlnB* and *GlnK*, as members of the PII protein family, act as sensors of cell nitrogen status (Coutts et al., 2002). At low concentrations of NH_4_^+^, *GlnK* is fully uridylylated, allowing AmtB to actively transport NH_4_^+^. Meanwhile, *GlnK*/*GlnB* signals to the GlnL and GlnG layers for regulation of gene *glnA* expression. At higher NH_4_^+^ concentrations, however, *GlnK* binds to *AmtB* and inactivates it, rendering it unable to transport NH_4_^+^ (Thomas et al., 2000; Arcondéguy et al., 2001). In M7-15, no complete two-component regulatory system genes were identified, but a nitrogen state sensor *GlnB*, and GlnR, functioning as a global regulator involved in nitrogen metabolism (Wang et al., 2014), were isolated, potentially regulating the transcription of *glnA* and may even simultaneously regulate nitrite reduction (Tiffert et al., 2008). Researchers have found that during cell growth, *glnA* is transcribed by different promoters depending upon the different nitrogen utilization pathways of cell growth (Kramer et al., 1996). And Wang et al. (2022) found that the response of bacterial communities to changes in survival conditions was mainly due to significant changes in the transcriptional patterns of different genes. Therefore, the difference in *glnA* transcription under ammonium or nitrate treatments contributes to the preference of strain M7-15 for nitrate.

### 4.3 Limitations and implications

This study advances our understanding of microbial nitrogen source utilization by combining the phenotypic growth characteristics of *Burkholderia* sp. M6-3 and *Arthrobacter* sp. M7-15 with whole-genome and transcriptomic analyses. The dynamic transcriptional response was elucidated by RNA sequencing, and the molecular mechanism of the nitrogen source preference strategy was revealed. While our study provides novel insights into microbial nitrogen preference mechanisms, we acknowledge several limitations in the experimental design. Firstly, the current work relies on pure culture experiments under controlled laboratory conditions, without considering the influence of environmental factors (such as pH) on the metabolism of strains (Fuess et al., 2019), nor can it fully summarize the complex interactions and environmental fluctuations existing in natural soil ecosystems (Zengler and Zaramela, 2018). Combining microcosm experiments or in-situ verification will help explore the metabolic differences of microorganisms towards ammonium N and nitrate N in complex environments, which may be the direction of our further research. Second, although three biological replicates were included for transcriptomic analysis, the use of pooled RNA samples (equal proportions of three replicates) may have averaged out subtle expression variations among individual samples, potentially masking strain-specific responses. However, GFOLD was selected for this study due to its unique strengths in handling single-sample data. Unlike traditional methods that rely on replicate-based variance estimation, GFOLD calculates a generalized fold change (GFC) using posterior distributions of log fold changes, which effectively accounts for uncertainty in single-replicate datasets (Feng et al., 2012). This approach has been extensively validated in studies with limited biological replicates (Huang et al., 2024; Sheikh et al., 2024), where GFOLD outperformed other methods in ranking DEGs under similar conditions. Notably, GFOLD’s conservative ranking of DEGs reduces false positives while maintaining biological relevance, which is critical for studies with sparse data. The researchers demonstrated the robustness of GFOLD in data analysis despite the constraint of single biological replicates (Sheikh et al., 2024). The coincidence between the transcriptome results and the inorganic nitrogen preference characteristics of the strains also proved the reliability of the results.

Despite these limitations, our study establishes a foundational framework for understanding strain-specific nitrogen preference and highlights key regulatory genes (e.g., *glnA*) that warrant further exploration. These findings provide critical guidance for designing targeted experiments to unravel the intricate interplay between microbial genetics and nitrogen cycling in agricultural soils.

## 5 Conclusion

In this study, we isolated two nitrogen-assimilating bacterial strains, *Burkholderia* sp. M6-3 and *Arthrobacter* sp. M7-15, from acidic dryland red soil. The conclusion highlights key genetic and pathway insights: The differential nitrogen source preferences (NH_4_^+^-N for M6-3, NO_3_^−^-N for M7-15) are underpinned by distinct gene regulatory patterns. Notably, the glutamine synthetase-coding gene *glnA* emerges as a critical factor. Its significant upregulation in M7-15, absent in M6-3, correlates with M7-15’s nitrate preference. Additionally, the global regulator *GlnR* is proposed as a potential modulator of nitrogen metabolism gene expression, potentially explaining the *glnA*-related discrepancies. While both strains share similar nitrogen metabolism pathways (involving genes like *Nrt*/*NrtA*, *nasAB*, *nirBD*, *AmtB*, and *GlnB*/*GlnK*), regulatory variations in key genes drive their divergent preferences. These findings identify *glnA* and regulatory elements like *GlnR* as pivotal in shaping nitrogen source utilization strategies. This work strengthens the theoretical foundation for understanding microbial nitrogen preference mechanisms and offers critical clues for screening functional microorganisms to enhance inorganic nitrogen use efficiency.

## Data Availability

The datasets presented in this study can be found in online repositories. The names of the repository/repositories and accession number(s) can be found here: https://www.ncbi.nlm.nih.gov/genbank/, PRJNA891540, PRJNA891352.

## Footnotes

^1^ http://blast.ncbi.nlm.nih.gov/Blast.cgi
