# Supplementary material for: Transcriptome analysis of nitrogen assimilation preferences in Burkholderia sp. M6-3 and Arthrobacter sp. M7-15
Source: Front Microbiol. 2025 Apr 7;16:1559884. doi: 10.3389/fmicb.2025.1559884 (PMC12010642; doi:10.3389/fmicb.2025.1559884)
Supplement: Supplementary file 1 [file Data_Sheet_1.docx]

Supplementary Material

**Transcriptome profiling reveals the differential assimilating preferences to ammonium or nitrate of *Burkholderia* sp. M6-3 and *Arthrobacter* sp. M7-15**

**Ran Liu^1,2†^, Hongyi Qin^1†^, Qian Wang^2^, Cheng Chu^1^, Yunbin Jiang^2^, Huan Deng^4^, Cheng Han^2,3^*, Wenhui Zhong^1,2,3^**

^1^College of Zhongbei, Nanjing Normal University, Danyang, Jiangsu, 212300, China

^2^Jiangsu Provincial Key Laboratory of Materials Cycling and Pollution Control, School of Geographical Sciences, Nanjing Normal University, Nanjing, China

^3^Jiangsu Center for Collaborative Innovation in Geographical Information Resource Development and Application, Nanjing, China

^4^School of Environment, Nanjing Normal University, Nanjing, China

^†^These authors contributed equally to this work and share first authorship

* Correspondence: Cheng Han
^🖂^chenghan@njnu.edu.cn

*** Correspondence:** Corresponding Author: chenghan@njnu.edu.cn

# Supplementary Data

Supplementary Material should be uploaded separately on submission. Please include any supplementary data, figures and/or tables.

Supplementary material is not typeset so please ensure that all information is clearly presented, the appropriate caption is included in the file and not in the manuscript, and that the style conforms to the rest of the article.

# Supplementary Figures and Tables

## Supplementary Figures


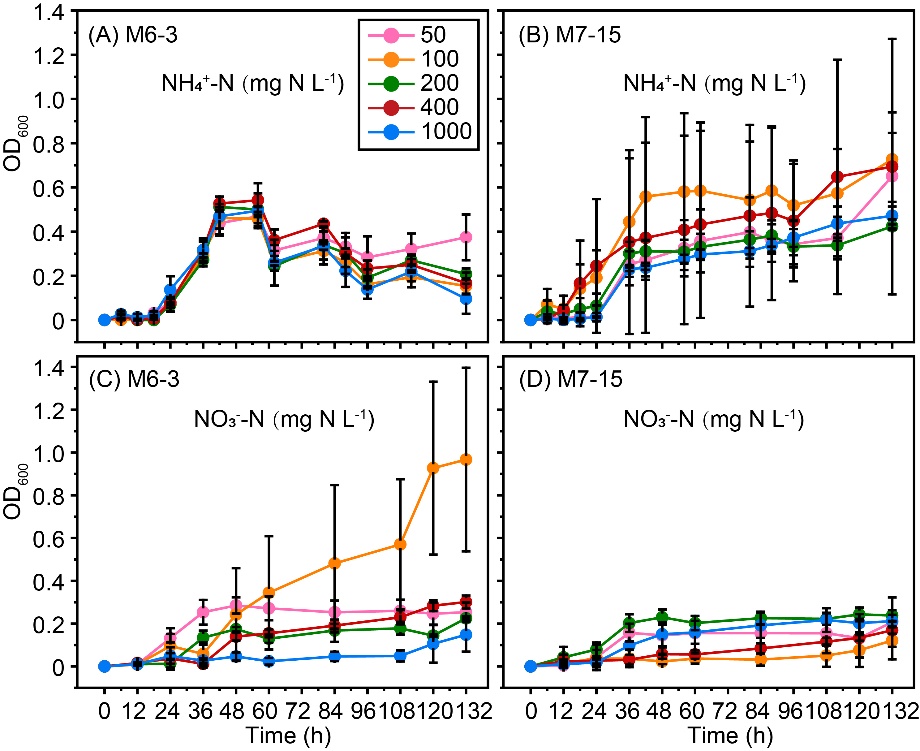


**Supplementary Figure 1.** Effects of different inorganic nitrogen concentrations on the growth of *Burkholderia* sp. M6-3 (A, C) and *Arthrobacter* sp. M7-15 (B, D).


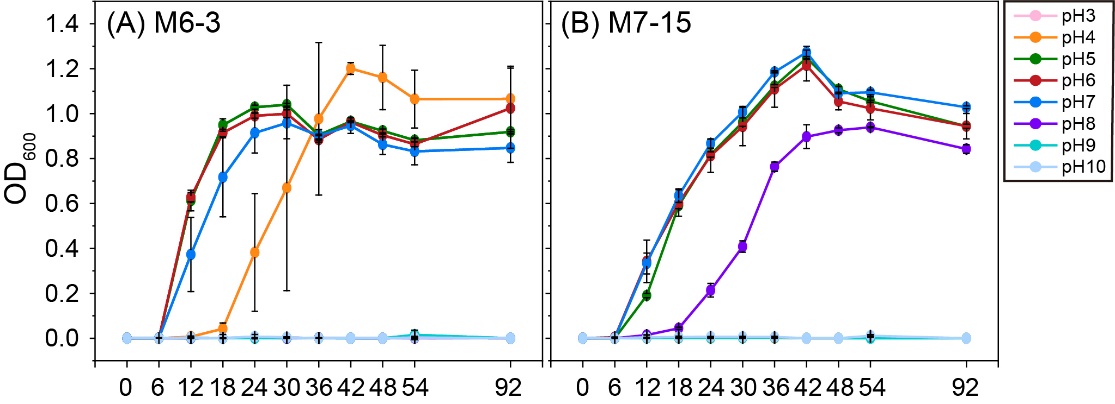


**Supplementary Figure 2.** Effects of different pH on the growth of *Burkholderia* sp. M6-3 (A) and *Arthrobacter* sp. M7-15 (B).


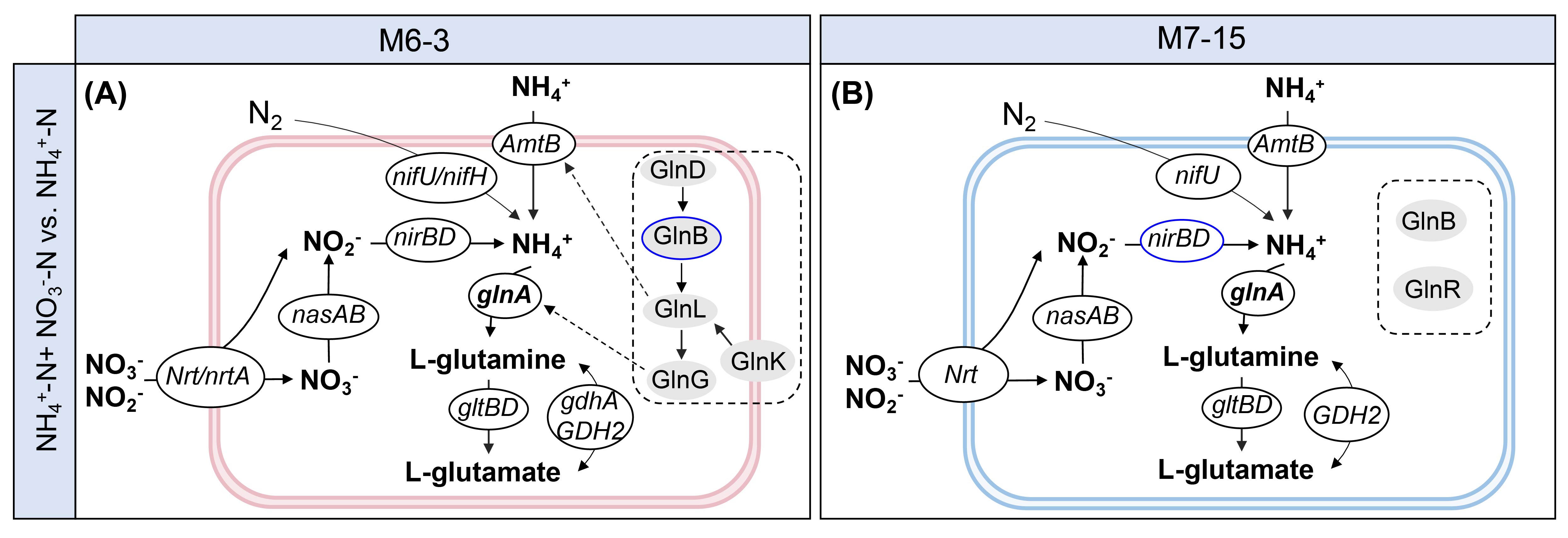


**Supplementary Figure 3.** Differential transcript expression of functional genes for nitrogen metabolism in sample groups of NH_4_^+^-N+NO_3_^-^-N vs. NH_4_^+^-N by *Burkholderia* sp. M6-3 and *Arthrobacter* sp. M7-15. Red borders indicate genes significantly up-regulated (Gfold > 1); blue borders indicate genes significantly down-regulated (Gfold < -1). Gray ovals indicate the nitrogen regulatory factors associate with the two-component regulatory system.

## Supplementary Tables

**Supplementary Table 1.** Genomic information and coding gene prediction of *Burkholderia* sp. M6-3 and *Arthrobacter* sp. M7-15.

| **Strain** | **M6-3** | **M7-15** |
| --- | --- | --- |
| Genome size (bp) | 8155157 | 4155715 |
| Gene number | 7989 | 4092 |
| Gene total length (bp) | 6812310 | 3655056 |
| Gene average length (bp) | 852.71 | 893.22 |
| Gene length/ genome (%) | 83.53 | 87.95 |
| Gene density (per kb) | 0.98 | 0.98 |
| sRNA gene number | 5 | 0 |
| rRNA genes number | 18 | 15 |
| tRNA gene number | 63 | 57 |

**Supplementary Table 2.** The number of total DEGs in different treatments of *Burkholderia* sp. M6-3 and *Arthrobacter* sp. M7-15.

| **M6-3** | **Gfold value** | **NO_3_^-^-N**  **vs. NH_4_^+^-N** | **NO_3_^-^-N+ NH_4_^+^-N vs. NH_4_^+^-N** | **NO_3_^-^-N+ NH_4_^+^-N vs. NO_3_^-^-N** |
| --- | --- | --- | --- | --- |
| Up-regulated | > 1 | 593 | 140 | 402 |
| Down-regulated | < -1 | 709 | 341 | 409 |
| **M7-15** | **Gfold value** | **NO_3_^-^-N**  **vs. NH_4_^+^-N** | **NO_3_^-^-N+ NH_4_^+^-N vs. NH_4_^+^-N** | **NO_3_^-^-N+ NH_4_^+^-N vs. NO_3_^-^-N** |
| Up-regulated | > 1 | 238 | 15 | 201 |
| Down-regulated | < -1 | 192 | 176 | 334 |
